# Supplementary material for: Influence of CO2 and Dust on the Survival of Non-Resistant and Multi-Resistant Airborne E. coli Strains
Source: Antibiotics (Basel). 2024 Jun 14;13(6):558. doi: 10.3390/antibiotics13060558 (PMC11201083; doi:10.3390/antibiotics13060558)
Supplement: Supplementary file 1 [file antibiotics-13-00558-s001.zip › antibiotics-3040703-supplementary.pdf]

- Supplementary Information –

Influence of CO_2 and Dust on the Survival of Non-resistant and Multi-resistant Airborne *E.coli* Strains

Agarwal, V.[1,2], Abd El, E.[3,4], Danelli, SG.[3,4], Gatta, E.[3], Massabo, D.[3,4], Mazzei, F.[3,4], Meier, B.[1], Prati, P.[3,4], Vernocchi, V.[4], Wang, J.[1,2*]

[1]Institute of Environmental Engineering, ETH Zurich, Zurich 8983, Switzerland

[2]Laboratory for Advanced Analytical Technologies, Empa, Swiss Federal Laboratories for Materials Science and Technology, Dübendorf 8600, Switzerland

[3]Dipartimento di Fisica – Università di Genova, via Dodecaneso 33, 16146, Genoa, Italy

[4]INFN – Sezione di Genova, via Dodecaneso 33, 16146, Genoa, Italy

* Correspondence: jing.wang@ifu.baug.ethz.ch

***S1:** Photographs of the atmospheric simulation chamber ChAMBRe at INFN, Genoa; a) overview of the chamber; b) automated shelf to insert and extract petri dishes into and out of the chamber; c) inlet valve for the bacterial injection*

a)

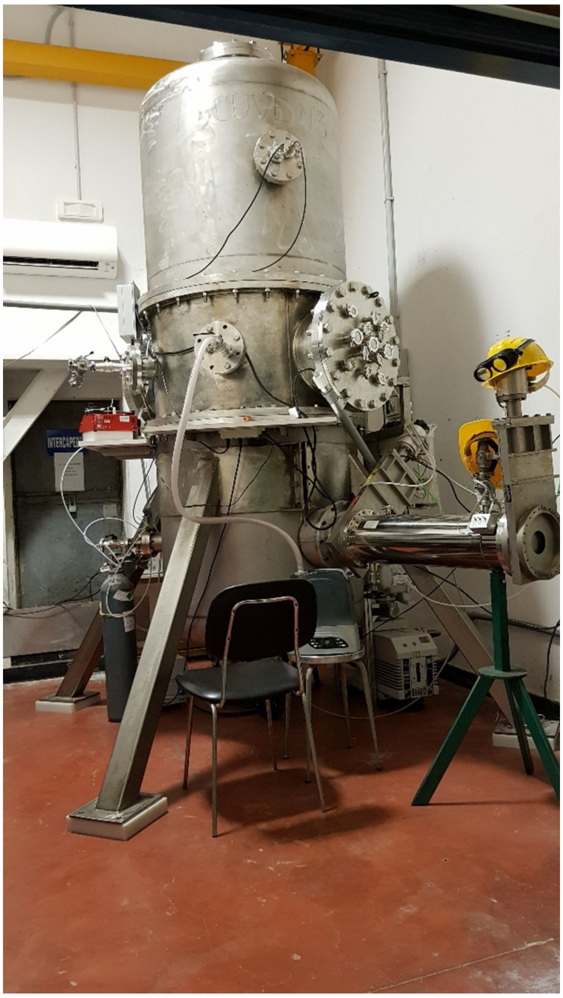

b)

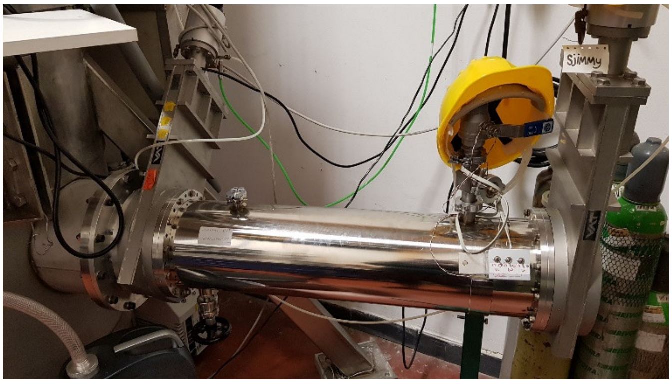

c)

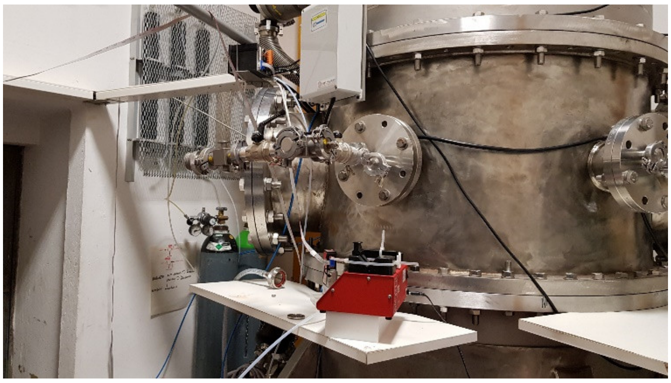

***S2:** Discussion about limitations and challenges faced during the conduction of the experiments*

During the experiments, we encountered issues with the bacterial strains exhibiting different behaviors after prolonged periods in the freezer and subsequent re-thawing. Occasionally, the intervals between experiments exceeded several months. Upon thawing out of the freezer, the bacteria were cultured on appropriate culture media as usual. However, we observed significantly slower growth in LB broth and discrepancies in results compared to previous experiments. In such instances, new baseline experiments were conducted, establishing a new baseline against which subsequent experiments with CO_2 and dust were compared. The slopes and R^2 values of the baselines are as follows:

- JM109 (Baseline 1): $y=470.5x$; $R^2=0.998$
- JM109 (Baseline 2) : $y=92.7x$; $R^2=1.000$
- JM109 (Baseline 3) : $y=25.5x$; $R^2=0.997$
- JM109-pEC958 (Baseline 1) : $y=248.9x$; $R^2=0.997$
- JM109-pEC958 (Baseline 2) : $y=111.6x$; $R^2=0.998$
- JM109-pEC958 (Baseline 3) : $y=38.1$; $R^2=0.959$

While the exact reasons for this behavior of the bacteria are not fully understood, it is likely related to the stress imposed on the cells during freeze-thaw cycles. Aside from the well-known cell damage during repeated freeze-thaw cycles, a previous study by Bruhn-Olszewska (2018) showed that extreme temperatures can induce dormancy in cells, rendering them non-dividing and non-growing (57,58). Consequently, freshly thawed cells may exhibit lower growth rate due to a higher ratio of dormant to dividing cells. Compared to previously cultured cells, re-thawed cells may contain a greater proportion of viable but non-culturable (VBNC) cells, necessitating the establishment of a new baseline.

When the new baselines were utilized, similar survival rates in percentage were obtained as in earlier experiments that were compared to the initial baseline. Therefore, all results were combined into the final results.

Furthermore, there were instances where no bacterial growth or uncountable growth was observed from chamber experiments, suggesting potential errors during bacterial suspension handling, chamber injection, or chamber settings. These experiments were excluded from the analysis.
